# Successful application of human-based methyl capture sequencing for methylome analysis in non-human primate models

**DOI:** 10.1186/s12864-018-4666-1

**Published:** 2018-04-18

**Authors:** Ja-Rang Lee, Dong-Sung Ryu, Sang-Je Park, Se-Hee Choe, Hyeon-Mu Cho, Sang-Rae Lee, Sun-Uk Kim, Young-Hyun Kim, Jae-Won Huh

**Affiliations:** 1 Primate Resource Center, Korea Research Institute of Bioscience and Biotechnology, Jeongeup, 56216 Republic of Korea; 2 Theragen Etex Bio Institute, Suwon, Republic of Korea; 3 National Primate Research Center, Korea Research Institute of Bioscience and Biotechnology, Cheongju, 28116 Republic of Korea; 4 Futuristic Animal Resource and Research Center, Korea Research Institute of Bioscience and Biotechnology, Cheongju, 28116 Republic of Korea; 5 Department of Functional Genomics, KRIBB School of Bioscience, Korea University of Science and Technology (UST), Daejeon, 34113 Republic of Korea

**Keywords:** Methyl-capture sequencing, DNA methylation, Animal model, Cynomolgus monkey, African green monkey

## Abstract

**Background:**

The characterization of genomic or epigenomic variation in human and animal models could provide important insight into pathophysiological mechanisms of various diseases, and lead to new developments in disease diagnosis and clinical intervention. The African green monkey (AGM; *Chlorocebus aethiops*) and cynomolgus monkey (CM; *Macaca fascicularis*) have long been considered important animal models in biomedical research. However, non-human primate-specific methods applicable to epigenomic analyses in AGM and CM are lacking. The recent development of methyl-capture sequencing (MC-seq) has an unprecedented advantage of cost-effectiveness, and further allows for extending the methylome coverage compared to conventional sequencing approaches.

**Results:**

Here, we used a human probe-designed MC-seq method to assay DNA methylation in DNA obtained from 13 CM and three AGM blood samples. To effectively adapt the human probe-designed target region for methylome analysis in non-human primates, we redefined the target regions, focusing on regulatory regions and intragenic regions with consideration of interspecific sequence homology and promoter region variation. Methyl-capture efficiency was controlled by the sequence identity between the captured probes based on the human reference genome and the AGM and CM genome sequences, respectively. Using reasonable guidelines, 56 and 62% of the human-based capture probes could be effectively mapped for DNA methylome profiling in the AGM and CM genome, respectively, according to numeric global statistics. In particular, our method could cover up to 89 and 87% of the regulatory regions of the AGM and CM genome, respectively.

**Conclusions:**

Use of human-based MC-seq methods provides an attractive, cost-effective approach for the methylome profiling of non-human primates at the single-base resolution level.

**Electronic supplementary material:**

The online version of this article (10.1186/s12864-018-4666-1) contains supplementary material, which is available to authorized users.

## Background

Because of their close evolutionary relationship with humans, non-human primates (NHPs) are considered valuable animal models for biomedical research [1]. NHPs show a high degree of similarity to humans in the genome sequence; e.g., 98.77% similarity with chimpanzee [2], 93.5% similarity with rhesus monkey [3], and 92.83% similarity with cynomolgus monkey [4]. In addition, NHPs share many physiological, immunological, and morphological similarities with humans. Moreover, they have numerous advantages as animal models for translation to humans, including controllability of environmental factors, ease of scale, and comparability of results [5]. Therefore, use of an NHP animal model could provide particularly valuable information in the development of vaccines and drugs, and for establishing preventive and therapeutic measures against emerging pathogens [6].

Among the NHPs, crab-eating or cynomolgus macaque (CM; *Macaca fascicularis*), rhesus macaque (*Macaca mulatta*), and African green monkey (AGM; *Chlorocebus aethiops*) are most commonly used for biomedical research [7]. These primates belong to the group of Old World monkeys, and diverged from the common ancestor of human and Old World monkeys about 32 million years ago [8]. Their close relationship to humans has made these primate species particularly suitable as animal models for biomedical research and evolutionary studies [9–12]. Rhesus macaques, of Indian origin, have served as a traditional animal model for human diseases [1]. However, since the export of rhesus macaques from India was banned in 1978, they have become harder to obtain. As an alternative, CM has been more widely adopted as an animal model for human disease. In addition, CM has several important advantages as an animal model compared to rhesus macaque: (1) easy handling due to its smaller body size and weight; (2) low cost and better availability for experimental use; and (3) lack of seasonal fertility [13]. AGM has long been considered an important animal model for biomedical applications such as in human immunodeficiency virus research, because they show resistance to simian immunodeficiency virus [5]. Recently, the draft genomes of AGM and CM were published, and the sequences are now available in various genomic databases (AGM GenBank Assembly ID, GCA_000409795.2; CM GenBank Assembly ID, GCA_000364345.1). Therefore, these primates could now serve as attractive animal models, and their contribution to biomedical research is expected to increase in the coming years.

DNA methylation, as an important epigenetic regulation, occurs at the 5-carbon residues of cytosine via the addition of a methyl group, which is catalyzed by DNA methyltransferases. In mammalian genomes, DNA methylation is predominantly found in CpG dinucleotides. In particular, methyl-cytosine is observed in up to 80% of normal human cells [14]. However, the occurrence of methylation is generally suppressed in GC-rich DNA, consisting of several regions known as CpG islands (CGIs). Approximately 60% of all known human genes are associated with CGIs in their promoter regions [15]. Methylation in the promoter region is closely associated with downstream gene silencing, and this modification not only regulates gene expression but also plays a role in numerous cellular processes, including X-chromosome inactivation, imprinting, embryonic development, maintenance of genomic stability, and transposon inactivation [16]. In somatic cells, DNA methylation patterns are stably maintained, and are inherited to daughter cells through mitotic cell division. However, they are not permanent. In fact, changes in DNA methylation are dynamically regulated during the mammalian life cycle [17]. In addition, changes in DNA methylation patterns are induced by several extrinsic factors derived from environmental exposure, ranging from a natural physiological response to environmental changes to those associated with the development of diseases such as neurodegenerative disorders, diabetes, cardiovascular disease, and various types of cancer [18]. Therefore, an aberrant DNA methylation change is a highly promising molecular biomarker for the early detection, diagnosis, and prognosis of complex or chronic diseases. For this reason, it is of great value to investigate the DNA methylome of AGM and CM as important animal models for human disease. However, establishment of a genome-wide approach to explore the DNA methylome of NHPs has thus far been hampered by the lack of suitable tools and cost limitations.

Many methods for genome-wide DNA methylation analysis at the single-base resolution are available for human samples, which can be divided into two main categories: microarray- and next-generation sequencing (NGS)-based methods. The microarray-based Infinium Human Methylation450 BeadChip Array (Infinium 450 K) has been widely used for epigenetics analyses owing to its advantages of cost-effectiveness, rapid sample processing time, and possibility for high-throughput processing of bulk samples [19]. However, the main limitation of microarray-based methods is the requirement for a fixed number of probes that target specific genome loci. Therefore, microarray-based methods are only suitable for screening a genome at known methylation-altered loci. Alternatively, NGS-based methods can be further refined according to the targeted genome regions. Whole-genome bisulfite sequencing (WGBS) is considered the gold-standard method, which can provide the highest genomic coverage and nucleotide resolution for quantification of DNA methylation [20]. However, this method is associated with substantial costs and a relatively long processing time for obtaining high-quality sequences, which have limited its widespread application. To reduce the associated sequencing costs and processing time, methyl-capture sequencing (MC-seq) is an attractive option, which allows for the selection of predefined genomic regions, and utilizes target-specific genomic loci of physiological and clinical interest [21]. The MC-seq method has various advantages of cost-effectiveness, broader genome coverage, and avoidance of the bias due to CpG-rich repeats. However, before the MC-seq method can be applied to NHPs, it is essential to first determine the applicability of the human genome-based captured probes for these models.

Toward this end, in this study, we sought to determine the applicability and accuracy of a human-based MC-seq kit to the AGM and CM genomes. We redefined the MC-seq target region for methylome analysis considering the probe sequence similarity and variation in the promoter regions of the same genes between human and NHPs. Adaptation of the established human MC-seq method for NHPs can be a powerful tool for epigenome analysis, and help provide novel information about DNA methylation alteration patterns with direct clinical translation.

## Methods

### Sample collection and extraction of primate genomic DNA samples

Ethical approval for collecting blood samples of cynomolgus macaques and African green monkeys was granted by the Institutional Animal Care and Use Committee (KRIBB-AEC-140007, KRIBB-AEC-15031 & KRIBB-AEC-15046) of the Korea Research Institute of Bioscience and Biotechnology (KRIBB). Animal preparation and study design were conducted according to the Guidelines of the Institutional Animal Care and Use Committee. Blood samples of cynomolgus macaques and African green monkeys were provided by the National Primate Research Center of Republic of Korea.

Genomic DNA samples were isolated from the peripheral blood of 13 specific pathogen-free female CMs (1–9 years old), one female AGM (20 years old), and one male AGM (16 years old), which were collected in each of the last 2 years for periodic health monitoring. Blood samples were collected by venipuncture and stored in PAXgene tubes (PreAnalytiX, Hombrechtikon, Switzerland). Genomic DNA was extracted using the PAXgene Blood DNA Kit (Qiagen, Hilden, Germany).

### Definition of targeted genomic regions

**Fig. 1:**
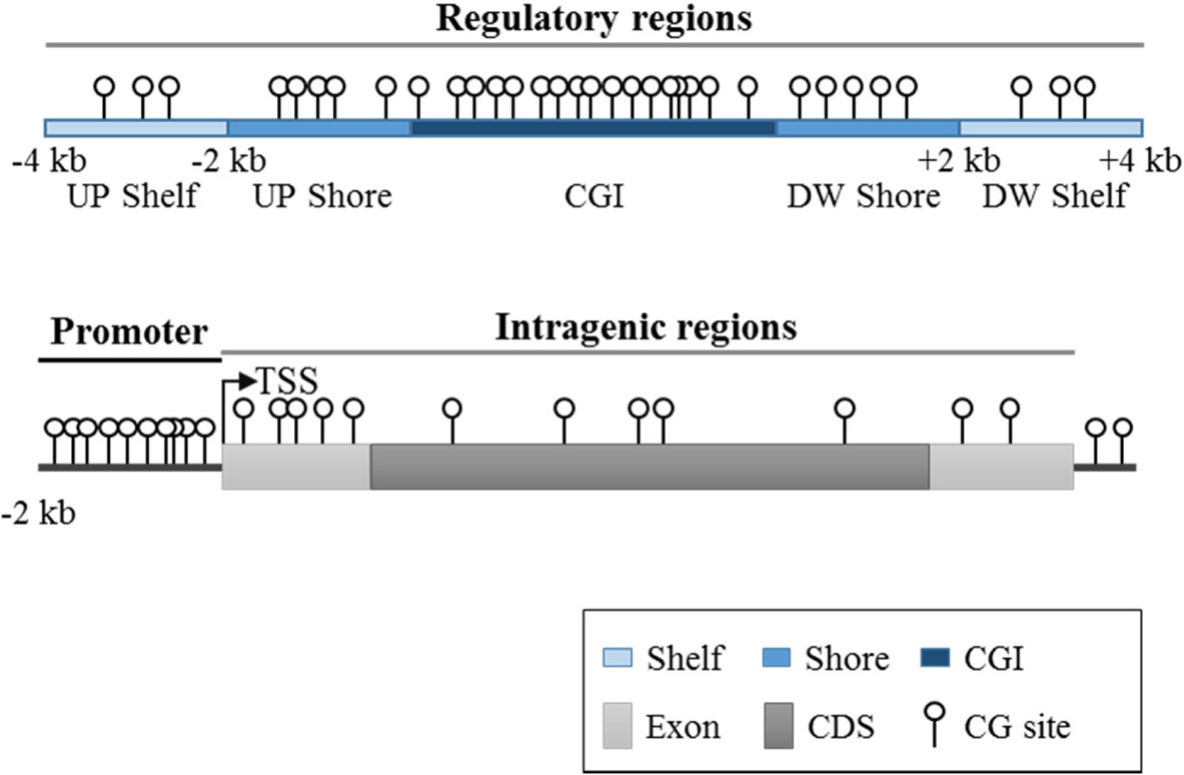
Definitions of various genomic regions used for methyl-capture sequencing in this study. Targeted genomic regions can be separated into regulatory regions and intragenic regions. Regulatory regions included CG islands (CGIs) with the surrounding CGI shore, CGI shelf, and promoter region. Intragenic regions are organized into all exon regions, including the coding sequence (CDS), excluding introns. US, upstream; DS, downstream; TSS, transcription start site

Targeted genomic regions were divided into regulatory and intragenic regions (Fig. 1). Regulatory regions contain promoters, CGIs, and CGI flanking regions (shore and shelf). As one of the most important regulatory regions, CGI regions were predicted by cpgreport, a widely used CGI prediction tool in the EMBOSS package, using default parameters [22]. The shore and shelf flanking regions were determined from the predicted CGIs, which span up to 2 kb from the end or start of the CGI and ≥ 2 kb from the end or start of the shore, respectively (Fig. 1) [23, 24]. In addition, promoter regions were defined to 2 kb upstream from the transcription start site (TSS) in present study. To determine the span of the promoter region, the TSS was calculated as the start site of the longest transcript among the transcripts associated with the same gene symbol. Ensembl 75 was used to calculate the promoter region and to define intergenic or intragenic genomic regions.

### Homologous probe region (HPR)

The NHP genome has a high level of sequence similarity to the human genome in view of the close evolutionary relationship. To identify the NHP target region, we extracted the human genome sequences located on the captured region by the probes of the SureSelect^XT^ Human Methyl-Seq (Agilent Technologies, Santa Clara, CA, USA). The extracted genome sequences were aligned to the NHP genome by the local alignment tool BLAT [25], which is particularly useful to align consecutive genomic sequences as much as possible. For blat parameters, we used default values which just consider DNA alignment (−q = dna; −out = blast8; −t = dna). Then, we selected the sequences based on the alignment options (identity and e-value) among the various aligned regions. The alignment results are summarized in Additional file 1: Table S1.

The homologous probe region has to be determined to allow for the efficient and precise use of the target genomic region with sufficient average sequence depth for confidence. In this study, we selected an identity value of 85% and an e-value of 1.0 × 10^− 10^, which allowed for the target region to reach a near-average depth of 30-fold for each CpG site, which is considered to be a reasonable depth for methylation analysis [26]. These values also permit sufficient use of the coverage in the SureSelect^XT^ Human Methyl-Seq (84 Mb) up to ~ 60%. We redefined the calculated target region determined using this approach as the HPR.

### Orthologous promoter region (OPR)

**Fig. 2:**
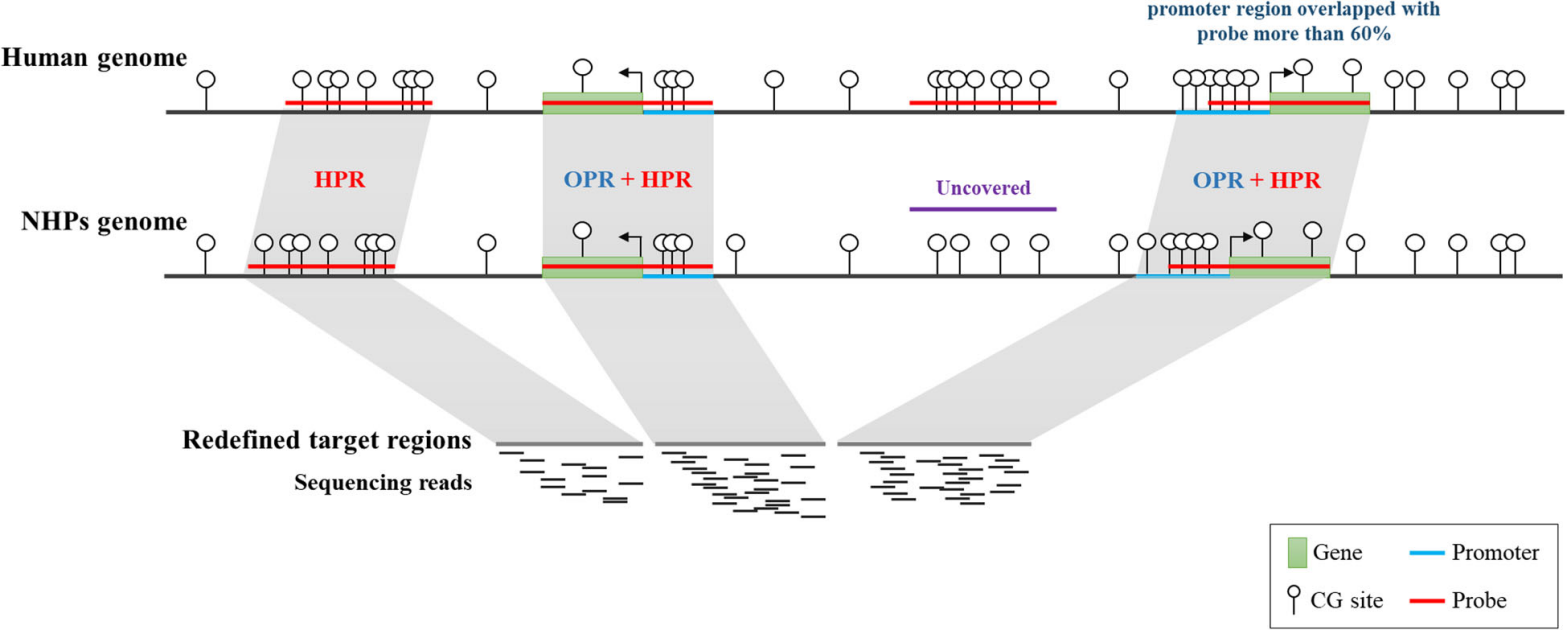
The redefined target region comprising the homologous probe region (HPR) and orthologous promoter region (OPR). To determine inter-species homologous genome sequences, we defined the HPR as alignment regions greater than the empirical thresholds (e-value and identity of the BLAT alignment result). For promoter regions not covered by the HPR, the OPR was also considered, consisting of the gene symbols that match between the non-human primate (NHP) genomes and human targets for which the promoter regions overlap with the probe targeted-region by more than 60%

In general, it is important to estimate the methylation level of CpG sites located on the promoter region from the perspective of gene regulation. Since probes of the human toolkit are designed to capture portions of the promoter regions, the whole promoter regions annotated in the Ensembl or UCSC databases, which are generally used for methylome analysis, are not targeted. Therefore, to compensate for this partial annotation and achieve a more expanded analysis of the uncovered regions that are not included by the sequence homology-based method, we added the OPR to the redefined target regions (Fig. 2). To define the captured gene symbols by the human tool kit, we listed the gene symbols that overlapped by more than 60% with the human targeted probe region. We then selected the gene symbols that matched with NHP gene symbols, and the NHP promoter regions were re-calculated to 2 kb in the 5′ direction from the TSS.

### Redefined target region

The CG distribution of the redefined target region corresponding to regulatory and intragenic regions is summarized in Additional file 2: Table S2. The redefined target region includes 1,680,406 (HPR, 1.66 million) and 1,812,429 (HPR, 1.81 million) CG sites in AGM and CM genome, and could cover 53.5% (HPR, 52.8%) and 57.7% (HPR, 57.6%) of the CG sites compared to human targeted CG sites, respectively. In this study, we redefined the new target region focusing on the sequence homology between the NHP and human genomes. To consider a more extended HPR, we could adjust the mismatch parameters to be more loose during the alignment (Additional file 1: Table S1).

### MC-seq and analysis

For NHP MC-seq analysis, DNA extracted from blood samples of three AGMs and 13 CMs were sequenced. We prepared the genomic libraries using the SureSelect^XT^ Methyl-Seq Target Enrichment System [27] for NHP MC-seq. The probes of this human toolkit are designed to capture 3.7 million CpG sites over an 84-Mb region, targeting DNA fragments of CG-rich regions (CGIs, including the shore and shelf), promoter regions, as well as known cancer- and tissue-specific differentially methylated regions (DMRs). All NHP samples were sequenced using the same workflow. In brief, genomic DNA was randomly sheared and then DNA fragments of 150–200 bp were extracted. The DNA fragments were subjected to end repair, adapter ligation, hybridization to SureSelect^XT^ Methyl-seq Capture Library, streptavidin bead enrichment, bisulfite conversion, and PCR amplification, and then unique index tags were added by PCR amplification. DNA sample libraries were sequenced with an Illumina Hiseq2000 sequencer according to the manufacturer’s instructions. The length of the sequenced read was 101 base pair-ends. For mapping of the sequenced reads, we used the reference sequences GCA_000409795.2 and GCA_000364345.1 for AGM and CM with the Ensembl 78 and Ensembl pre-version annotation databases, respectively. In the case of the CM reference, the Ensembl and NCBI databases could not provide sufficient annotation information for methylome analysis, since this reference is a pre-assembled version. The human reference genome sequence hg19 in the UCSC database was used for comparison or analysis with the Ensembl 75 annotation database. For mapping of bisulfite-converted reads, we used *Bismark* [28], which provides the minimized bias result by using a best-hit alignment strategy. In order to improve the accuracy of bisulfite alignment, we designated -N parameter as 0 (maximal mismatches permitted). At the case of other parameters, default values were used. The same version of the *Bismark* package was used for uniquely mapped sequences to the reference, de-duplication, and cytosine calling.

## Results

### Redefined target region for MC-seq analysis using a human probe capture system in the AGM and CM genomes

**Fig. 3:**
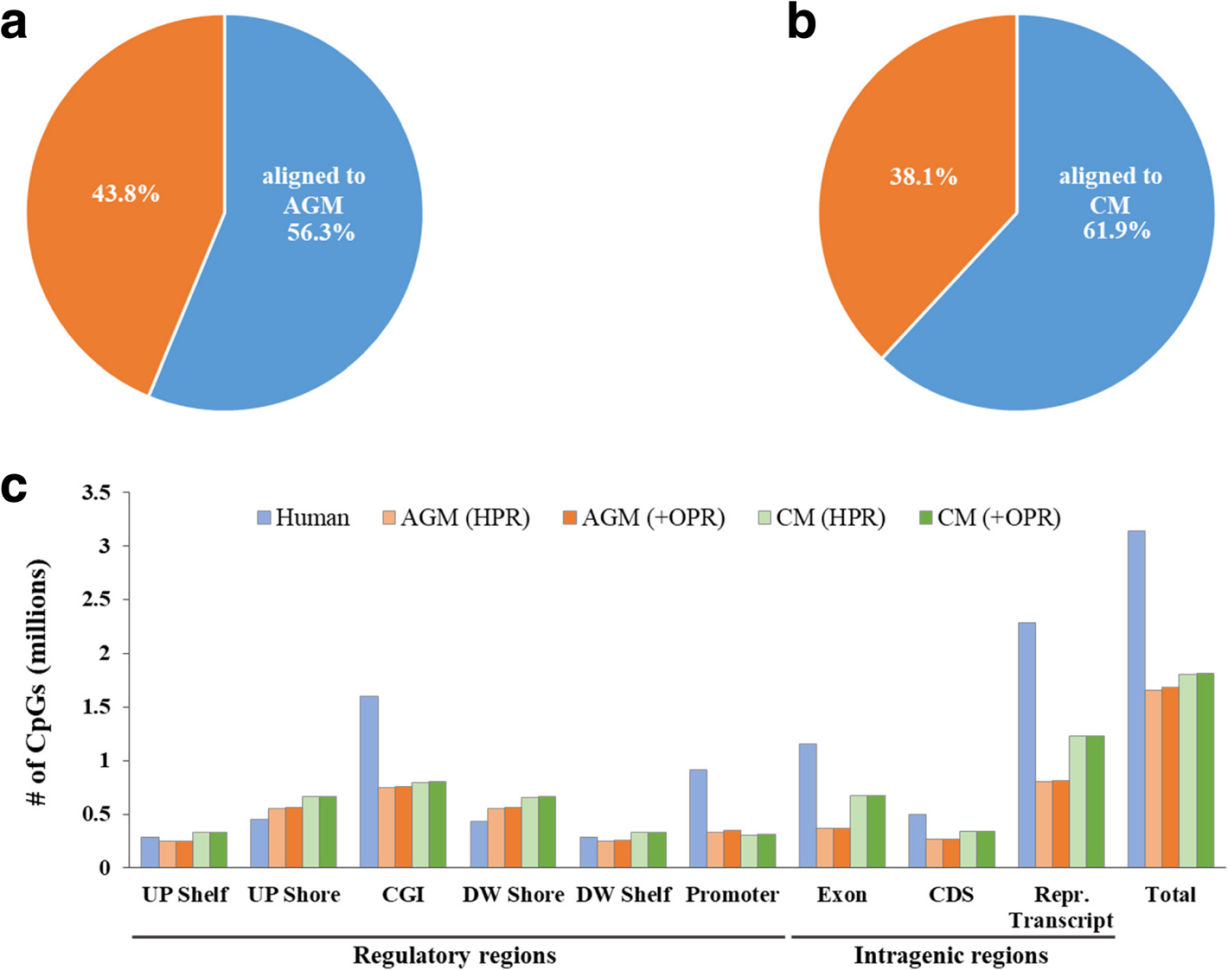
CpG content distribution according to each genomic region in redefined targeted regions. The pie charts show the percentages of sequences identified with the SureSelect human toolkit that could be aligned to the **a** African green monkey (AGM) and **b** cynomolgus macaque (CM) genome with an identity threshold of 85% and an e-value < e^− 10^. **c** The height of bar graphs represents the number of CpGs covered by the homologous promoter region (HPR) and orthologous promoter region (OPR) methods

To employ the human methyl-captured toolkit for NHPs, we searched the AGM and CM genome sequences with the human capture probe sequences using BLAT with an identity cut-off of 85% and an e-value threshold of 1.0 × 10^− 10^. Overall, 56.3 and 61.9% of all human capture probes could be successfully mapped to the AGM and CM genome, respectively (Fig. 3a, b; see Additional file 1: Table S1 for details). To further investigate how the redefined target regions are constructed on each annotated genomic region, we analyzed the CG site distribution according to the annotated genomic region (Fig. 3c). For comparison with AGM and CM, we also calculated the CG site distribution of the human toolkit on the human genomic region, respectively. The redefined regions covered 349,499 (HPR, 326,734) and 309,205 (HPR, 305,570) CG sites of promoter regions in AGM and CM (Additional file 2: Table S2). This coverage shows that the human target region overlapped with 67.8 and 58.1% of regions in the AGM and CM genome, respectively. Another encouraging fact was that the redefined regions could cover 752,032 (HPR, 747,884) and 798,668 (HPR, 796,929) of the CGI regions in AGM and CM (Additional file 2: Table S2). This extent of coverage corresponds to 88.6 and 86.5% of the coverage for the human targeted CGI regions. The additional OPR expanded 22,765 and 3635 CG sites in total for the targeted regions in AGM and CM, respectively (Additional file 2: Table S2). The OPR for CM could not be extended or covered more than that of AGM since the CM annotation database (pre-version) is not as well established as the AGM annotation database with respect to gene symbols. Thus, addition of the OPR appears to be more effective when dealing with well-established genomes; nevertheless, use of the OPR allowed for more expanded analysis of the uncovered regions in both the AGM and CM genomes.

### Evaluation of human-based MC-seq performance for AGM and CM samples

**Fig. 4:**
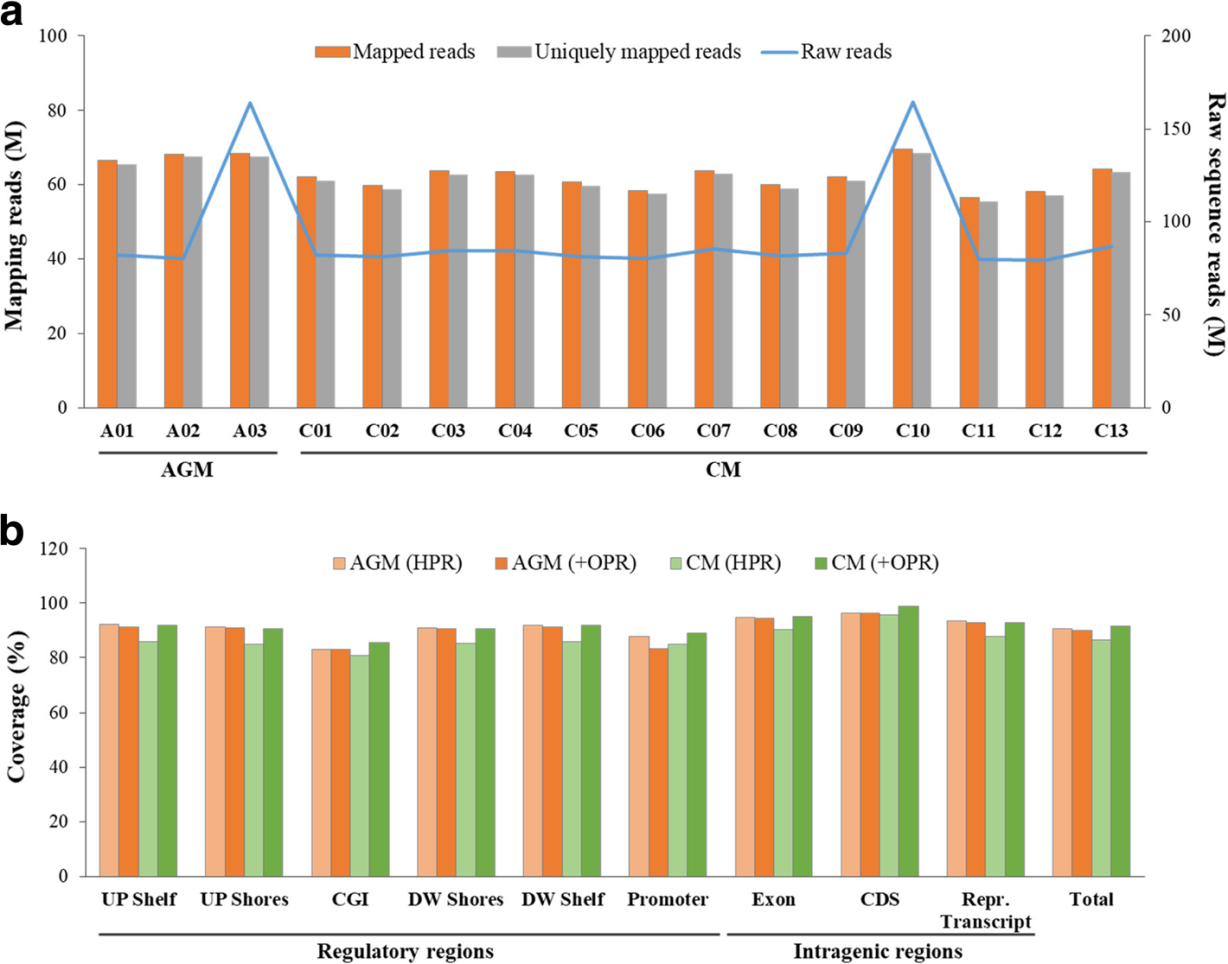
Read statistics. **a** Bars show the numbers of mapped reads and uniquely mapped reads, and the line represents the number of raw reads. Each vertical axis shows the number of reads in millions. **b** Bars represent the rate of coverage of on-targeted read by genomic regions at a calling depth ≥ 5-fold. AGM, African green monkey; CM, cynomolgus macaque; HPR, homologous promoter region; OPR, orthologous promoter region

The DNA methylomes of AGM and CM samples were generated using MC-seq with a SureSelect capture system and bisulfite-conversion approach. The mapping statistics are summarized in Fig. 4a and Additional file 3: Table S3. On average, 82 million pair-end reads were generated per sample, 62 million of which aligned uniquely to the bisulfite-converted AGM and CM genome. We sequenced each sample up to nearly 100-fold as a goal to acquire a sufficient amount of reads (more than 40-fold depth after de-duplication) that could then be used in the methylation-level calling. In the case of samples A03 and C10, we conducted additional sequencing to satisfy our criteria to acquire a sufficient amount of de-duplicated reads. The numbers of mapped reads between samples relative to the corresponding reference were similar, and the ratio of mapped reads was greater than 70%. After removing multiply mapped reads on the genome, the ratio of uniquely mapped reads in most of the samples was also greater than 70%, indicating that the sequencing data was of good quality. The protocol for the MC-seq method might be accompanied by a high level of duplicated reads caused by doubling of the PCR amplification. Therefore, we overcame this problem by adopting an acquisition strategy for de-duplicated reads. The average proportion of duplicated read was 25% for the primate genomes. Finally, we could secure the de-duplicated reads from more than 39 million reads of each sample, which could then be used for actual cytosine calling analysis (Additional file 3: Table S3).

**Fig. 5:**
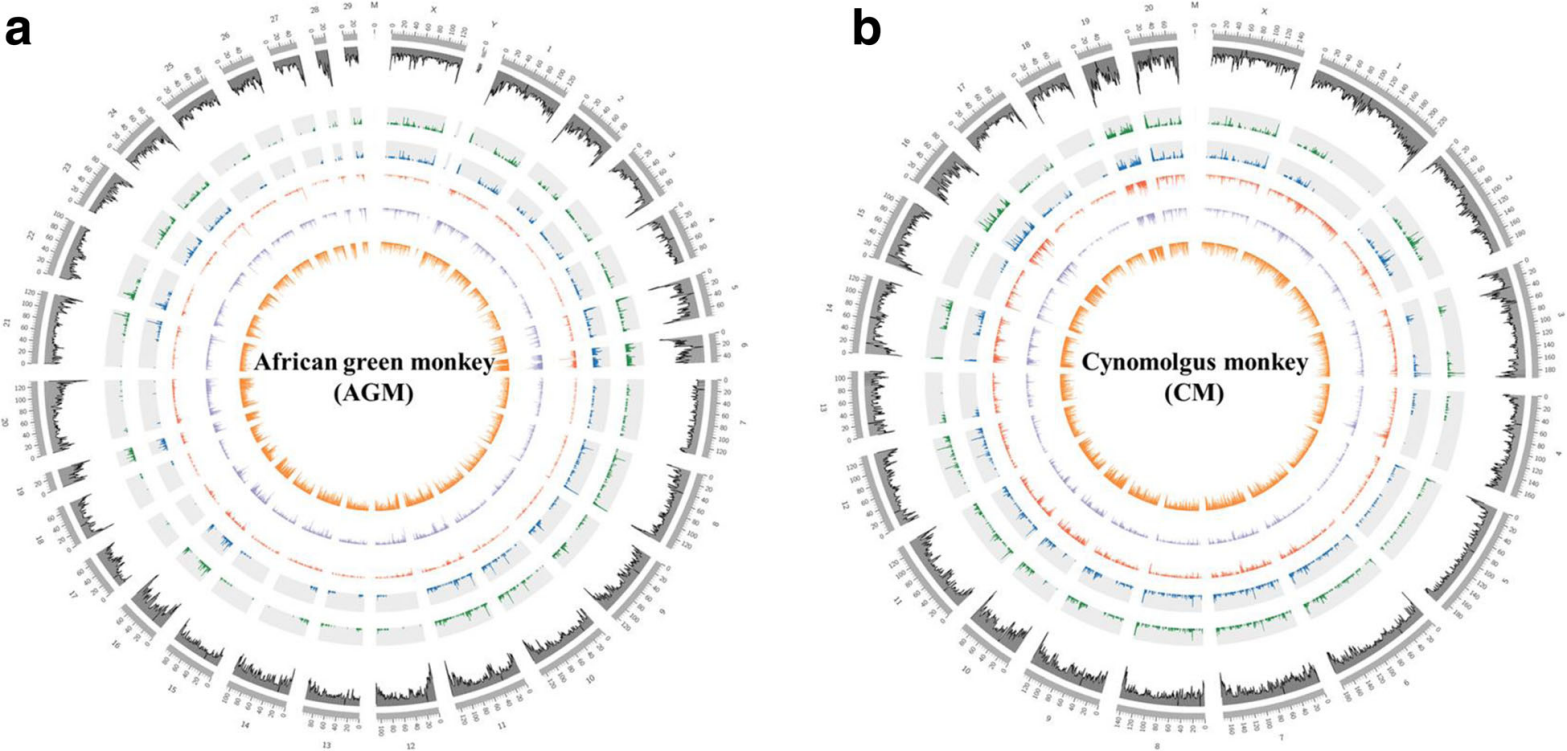
Comprehensive distribution maps of redefined targeted regions in African gene monkey (**a**) and cynomolgus macaque (**b**). Each numerical distribution according to the genomic region was calculated against a 500-kb bin on the whole genome. Black peaks, distribution of CpG sites on the whole genome; green peaks, distribution of target homologous promoter regions (HPRs) + orthologous promoter regions (OPRs) greater than 500 bp; blue peaks, distribution of target HPRs greater than 500 bp; red peaks, distribution of the representative transcript region; purple peaks, distribution of the promoter region; orange peaks, distribution of CGIs and flanking regions

After the alignment process, we filtered out the only on-targeted reads using the redefined target region as a guide. The ratio of on-targeted reads compared with de-duplicated reads ranged from 50.6 to 62.1%. The average ratio of on-targeted reads for the HPR and for the HPR plus OPR analysis was 59.8 and 59.9%, respectively. The average depths for the targeted regions with their cumulative percentages of CG sites according to depth in the target region are summarized in Additional file 4: Table S4. The cytosine calling depths were greater than 40-fold in all cases, except for sample A03. Furthermore, more than 90% of the targeted CG sites were covered at a ≥ 5-fold calling depth (Fig. 4b). These statistics show that our target region could provide adequate detailed resolution and capture performance at the on-targeted region to effectively estimate the methylation level using the human toolkit. If we set a more extended OPR with a loose overlap ratio of gene symbols using the human toolkit, we could obtain a greater number of on-targeted reads; however, this would come at a cost of low depth coverage of on-target reads. Finally, based on our criteria for a redefined target region, the comprehensive distribution maps of the target regions in the AGM and CM genomes were obtained (Fig. 5).

### Characterization of methylation levels in AGM and CM models

**Fig. 6:**
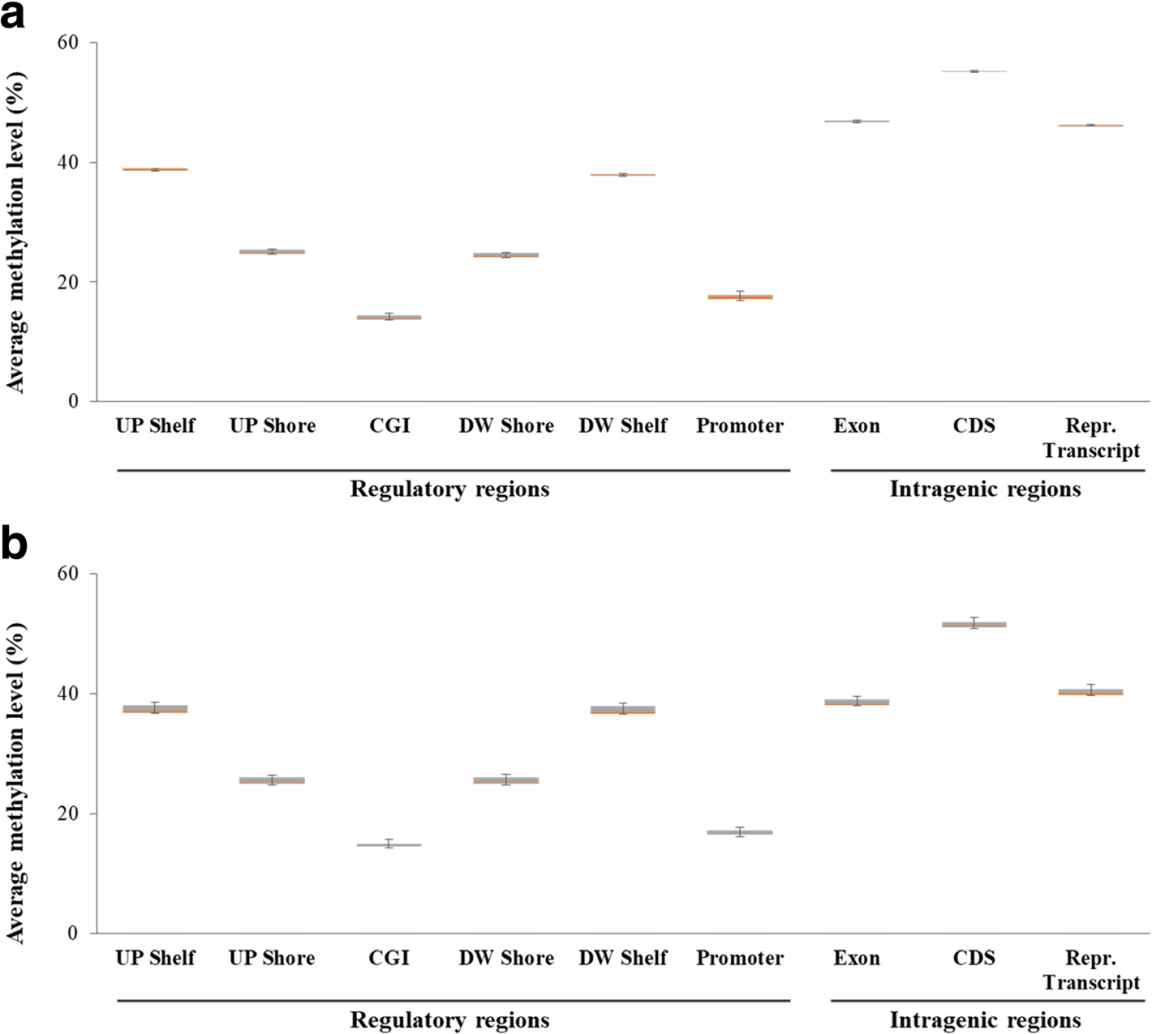
Average methylation levels according to each genomic region in the African green monkey (**a**) and cynomolgus macaque (**b**) genomes. The boxes and error bars indicate the mean and 95% confidence intervals, respectively

To confirm the accurate detection of methylome status using the human toolkit for analysis of AGM and CM samples, we investigated methylation levels with various genomic regions based on our MC-seq data (Fig. 6). Additional file 5: Table S5 shows the average methylation levels with their standard deviations for each genomic region. For CG methylation level estimation, we merged each strand of DNA. In the mammalian genome, intergenic DNA, exon regions, and transposable element sequences commonly show high methyl-cytosine levels [29], whereas the promoter and CGI regions are usually hypomethylated compared with the intragenic regions. Furthermore, CGIs and CGI flanking regions (including the shore and shelf) have been reported to show gradual hypermethylation patterns from the CGIs to the outside regions [30]. We confirmed these general global methylation patterns in both the AGM and CM genomic regions (Fig. 6). These results confirmed the reliable performance of the human-based MC-seq method applied to NHP genomes for global methylation analysis.

## Discussion

**Fig. 7:**
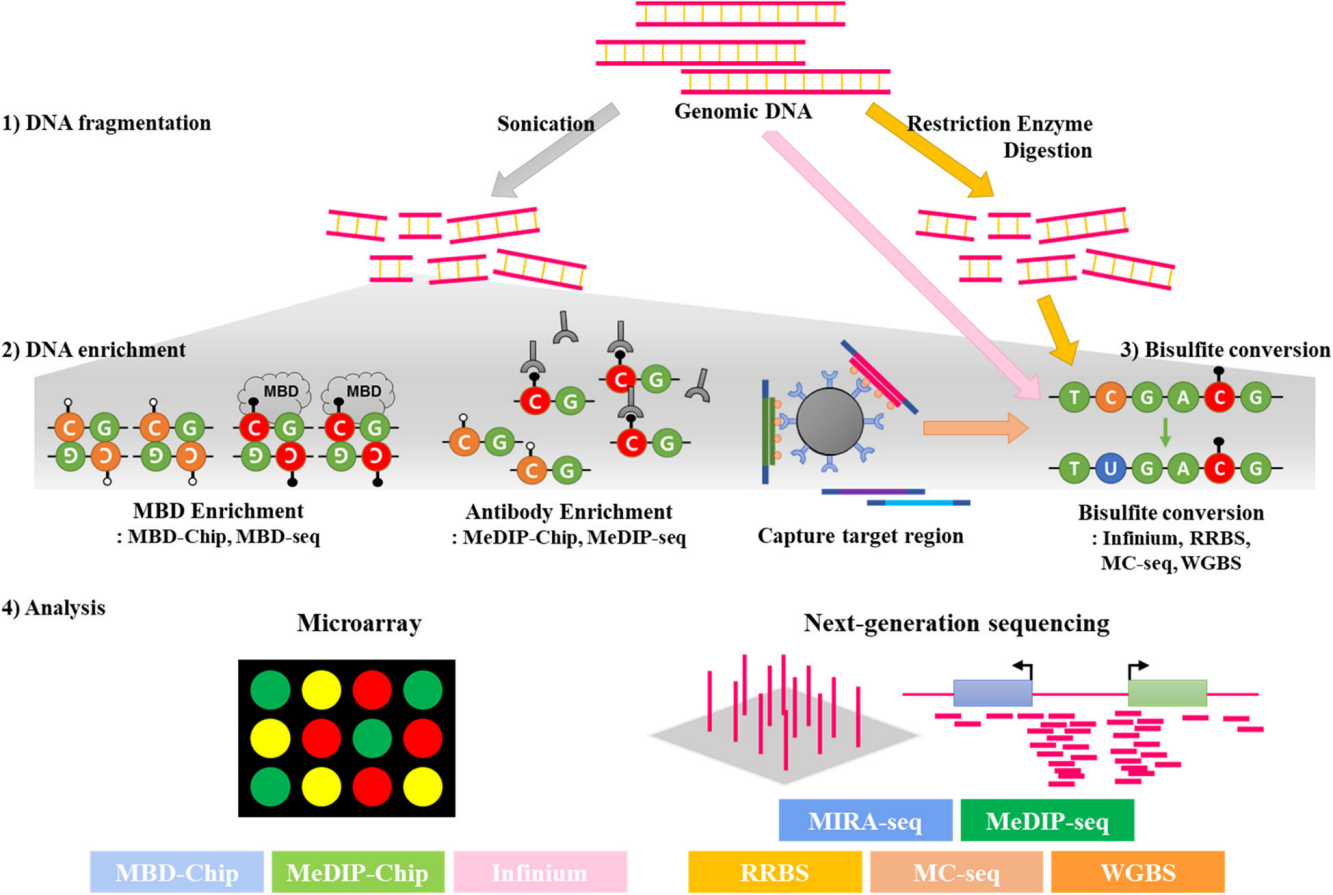
Schematic of the procedures for genome-wide DNA methylation analysis. 1) Fragmentation of genomic DNA by sonication or restriction enzyme digestion. 2) Target genomic DNA enrichment using MBD protein, methylation antibody, or target probe. 3) Bisulfite conversion or 4) direct genome-wide DNA methylation analysis by microarray or a next-generation sequencing platform. MBD, methyl-CpG-binding domain; MeDIP, methylated DNA immunoprecipitation; Infinium or HM450, Illumina, Infinium Human Methylation 450 BeadChips; RRBS, reduced-representation-bisulfite-sequencing; MC-seq, methyl-capture sequencing; WGBS, whole-genome bisulfite sequencing

With the development of microarray hybridization and NGS technologies, researchers must now consider several factors for genome-wide DNA methylation analysis according to the specific research purpose, including the available DNA amount, coverage, resolution, cost, and analysis terms. To facilitate appropriate selection of a genome-wide methylation platform for application to NHP models according to research needs, we classified all of the approaches available into microarray and NGS platforms, and comparatively subdivided the main methods with their details to serve as a guide (Fig. 7 and Table 1).

**Table 1:** Summary of experimental approaches for genome-wide DNA methylation profiling

	^a^Species	^b^DNA amount (μg)	Reads	Genome Coverage (CpG)	^c^Influencing factor	Resolution	Bioinformatics requirement	Cost
Array-based methods^d^								
MeDIP-chip	limited	5	–	depends on array	A, B, C, D	~ 150 bp	++	++
MBD-chip	limited	5	–	depends on array	A, B, C, D	~ 150 bp	++	++
Infinium	limited	0.5–1	–	485 K	C, E, G	Single base	+	+
NGS-based methods^e^								
MeDIP-seq	any	0.3–5	50 M	~ 23 M	A, B, D	~ 150 bp	+++	++
MBD-seq	any	1–3	30 M	~ 23 M	A, B, D	~ 150 bp	+++	++
RRBS	any	0.01–2	10 M	~ 2 M	E, F, G	Single base	+++	++
MC-seq	limited	1–3	50 M	3.7 M	E, G	Single base	**+++**	**++**
WGBS	any	1–5	> 500 M	> 28 M	E, G	Single base	+++++	+++++

*Abbreviations*: *MeDIP* methylated DNA immunoprecipitation, *MBD* methyl-CpG-binding domain, *NGS* next-generation sequencing, *RRBS* reduced-representation-bisulfite-sequencing, *MC-seq* methyl-capture sequencing, *WGBS* whole-genome bisulfite sequencing, *M* million, *K* thousand, + very low, ++ low, +++ moderate, ++++ high, +++++ very high

^a^Species: the range of applications varies according to the methylome profiling method adopted; methods are limited to species with commercially available arrays, or species with a complete reference genome available

^b^DNA input varies depending on the protocol

^c^Influencing factors represent the potential sources of genomic region bias. A, CG content; B, CpG density; C, probe hybridization; D, copy number variation; E, bisulfite conversion rate; F, enzyme recognition sites; G, bisulfite PCR bias

^d^References: MeDIP-Chip [39, 40]; MBD-Chip [40, 41]; Infinium [19, 34, 42–44]

^e^References: MeDIP-seq [32, 34, 45]; MBD-seq [33, 34, 44, 46]; RRBS [26, 34, 44, 47, 48]; MC-seq [21, 26, 34]; WGBS [20, 26, 34, 44, 49]

Although the WGBS method is considered to be the gold standard for genome-wide DNA methylation profiling, it is unsuitable for methylome screening or comparative profiling for diverse applications owing to the high cost and long processing time. The methylated DNA immunoprecipitation (MeDIP) method is easy to apply to any other species, including primates [31], because of the use of methylation-specific antibodies in the DNA enrichment process. However, the critical weak point of MeDIP-chip or MeDIP-seq is the resolution, which hinders methylation quantification at single-nucleotide resolution [32]. Methyl-CpG-binding domain (MBD)-chip or MBD-seq also allows for obtaining broader coverage of the genome, but these methods are also associated with a low resolution problem [33].

To reduce the cost and processing time at the single-base resolution, Infinium 450 K and MC-seq were suggested as reasonable alternatives to a WGBS platform for clinical DNA methylome studies or epigenome-wide association studies [34]. However, MC-seq appears to be a more attractive alternative platform for methylome analysis at the single-base resolution for large-scale analyses of clinical samples with respect to coverage, technical variation, and concordance of methylation calls [35]. A previous study showed that the Infinium 450 K array method could be accurately applied as a cross-species analysis of the DNA methylome of CM muscle tissues [36]. However, the suitability of MC-seq for NHP models has not been assessed to date. Here, we show that the redefined target region provided sufficient resolution (≥40-fold), and intermediate wide-coverage (≥56 Mb coverage) compared with other methylome analysis methods as Infinium 450 K [36] and WGBS [37]. Thus, we provide the first demonstration that human-based MC-seq is a practical and valuable approach for analyses of primate models, specifically in AGM and CM.

In this study, the SureSelect human toolkit from Agilent Technologies was used for the target enrichment of the AGM and CM genomes. This toolkit was designed for various target regions, including DNA fragments of CG-rich regions (CGIs, and shore and shelf regions), promoter regions, Refseq genes, Ensembl regulatory features, as well as known cancer- and tissue-specific DMRs on the human genome. Therefore, this MC-seq method is useful for analyses of the methylome. Based on this feature, we expect that our redefined target region might provide basic methylome data from an NHP model. Furthermore, the coverages for the AGM and CM genomes were similar to those obtained with the previous study using the Infinium 450 K array in CM: the human Infinium 450 K probes could cover approximately 61% of the designed regions in the CM genome [36], and the SureSelect human toolkit achieved almost 60% coverage of the designed regions in the AGM and CM genomes. Therefore, considering the genomic coverage of MC-seq (1.7–1.8 million CG sites), our results suggest that the SureSelect human toolkit can be applied to methylome analysis for an intermediate genomic range between that obtained with the Infinium 450 K (298,070 CG sites) [36] and WGBS (21 million CG sites) [37] platforms, applicable for NHP models. Further development and application of human-based MC-seq with NHP models should enable reasonable and powerful methylome screening or profiling analyses in various research fields with numerous advantages, including low cost, low bioinformatics requirements, high resolution, negligible interference of influencing factors, high genomic coverage, and requirement of a low sample amount.

## Conclusion

We demonstrated the applicability and accuracy of human-based MC-seq to assay the DNA methylome in blood samples collected from three AGMs and 13 CMs. We adapted the human MC-seq protocol to bisulfite sequencing for NHPs considering inter-species sequence homology and promoter region similarities. The redefined target region provides sufficient resolution (average 47-fold) to analyze the NHP methylome data. Although our method can only make use of 60% of the human probe-designed target region, it provided genome-wide coverage (1.7–1.8 million CG sites) that is intermediate between that obtained with the Infinium 450 K (298,070 CG sites) [36] and WGBS (21 million CG sites) platforms [37]. Human-based MC-seq has cost, and time effectiveness than WGBS, and has high performance than Infinium 450 K at the single-base resolution. In the human genome, the targeted probe region includes the cancer- and tissue-specific DMRs, CGIs, Gencode promoters, DMRs or regulatory features in CGIs, shores and shelves, DNase I hypersensitive sites, Refseq genes, and Ensembl regulatory features [38]. Our method can also capture the bisulfite sequences on the NHP genome that target the above-mentioned regulatory regions. Therefore, we conclude that human-based MC-seq can be a suitable approach for DNA methylome profiling of NHP animal models.

## Additional files

Additional file 1: **Table S1**.The length (Mb) of aligned homologous probe region according to identities and e-value. (DOCX 27 kb)

Additional file 2: **Table S2**. CG site distribution according to the genomic region in the redefined target region. (DOCX 28 kb)

Additional file 3: **Table S3**. Summary of alignment statistics about sequenced reads. (DOCX 28 kb)

Additional file 4: **Table S4**. On-targeted reads and average depth with accumulative depth coverage on CG sites. (DOCX 31 kb)

Additional file 5: **Table S5**. Average methylation level on CG sites for each genomic regions (mean ± s.d.). (DOCX 27 kb)

## Abbreviations

AGM: African green monkey
CGI: CpG island
CM: Cynomolgus macaque
DMR: Differentially methylated region
HPR: Homologous probe region
MBD: Methyl-CpG-binding domain
MC-seq: Methyl-capture sequencing
MeDIP: Methylated DNA immunoprecipitation
NGS: Next-generation sequencing
OPR: Orthologous promoter region
TSS: Transcription start site
WGBS: Whole-genome bisulfite sequencing
