# Supplementary material for: Successful application of human-based methyl capture sequencing for methylome analysis in non-human primate models
Source: BMC Genomics. 2018 Apr 18;19:267. doi: 10.1186/s12864-018-4666-1 (PMC5907189; doi:10.1186/s12864-018-4666-1)
Supplement: Supplementary file 1 — Table S1.The length (Mb) of aligned homologous probe region according to identities and e-value. (DOCX 27 kb) [file 12864_2018_4666_MOESM1_ESM.docx]

Table S1.The length (Mb) of aligned homologous probe region according to identities and e-value.

| **Species** | **Identity** | **E-value** $\boldsymbol{(1.0\times}\boldsymbol{10}^{\boldsymbol{-x}}\boldsymbol{)}$ | | | | | |
| --- | --- | --- | --- | --- | --- | --- | --- |
|  |  | $\boldsymbol{x=50}$ | $\boldsymbol{x=30}$ | $\boldsymbol{x=10}$ | $\boldsymbol{x=7}$ | $\boldsymbol{x=5}$ | $\boldsymbol{x=2}$ |
| AGM | 95 | 15.40  (18.34 %) | 16.77  (19.98 %) | 17.60  (20.97 %) | 17.73  (21.12 %) | 17.82  (21.22 %) | 17.96  (21.39 %) |
|  | 90 | 37.70  (44.91 %) | 41.47  (49.39 %) | 43.47  (51.78 %) | 43.74  (52.10%) | 43.88  (52.27%) | 44.07  (52.50 %) |
|  | **85** | 40.01  (47.66 %) | 44.58  (53.11 %) | **47.22**  **(56.25 %)** | 47.56  (56.65%) | 47.73  (56.85%) | 47.95  (57.12 %) |
|  | 80 | 40.24  (47.93 %) | 45.02  (53.62 %) | 47.92  (57.08 %) | 48.28  (57.51 %) | 48.47  (57.73 %) | 48.70  (58.01 %) |
| CM | 95 | 18.40  (21.92%) | 19.96  (23.77%) | 20.87  (24.86 %) | 21.01  (25,03%) | 21.10  (25.13 %) | 21.25  (25.31 %) |
|  | 90 | 42.14  (50.20%) | 46.18  (55.00 %) | 48.29  (57.52 %) | 48.57  (57.86%) | 48.73  (58.04%) | 48.93  (58.28 %) |
|  | **85** | 44.35  (52.83 %) | 49.20  (58.60 %) | **51.97**  **(61.88 %)** | 52.30  (62.30%) | 52.48  (62.51 %) | 52.71  (62.79 %) |
|  | 80 | 44.58  (53.10 %) | 49.64  (59.12 %) | 52.66  (62.73 %) | 53.04  (63.18%) | 53.23  (63.41%) | 53.48  (63.70 %) |

The percentages in parentheses mean the length ratio between HPR and the already designed region on human genome by Agilent Sureselect^XT^ (almost 83 Mb).
