# Supplementary material for: Successful application of human-based methyl capture sequencing for methylome analysis in non-human primate models
Source: BMC Genomics. 2018 Apr 18;19:267. doi: 10.1186/s12864-018-4666-1 (PMC5907189; doi:10.1186/s12864-018-4666-1)
Supplement: Supplementary file 2 — Table S2. CG site distribution according to the genomic region in the redefined target region. (DOCX 28 kb) [file 12864_2018_4666_MOESM2_ESM.docx]

Table S2. CG site distribution according to the genomic region in the redefined target region.

| **Species** | **Regulatory regions (# of CG sites/ percentage)** | | | | | |  | **Intragenic regions** | | | **Total** |
| --- | --- | --- | --- | --- | --- | --- | --- | --- | --- | --- | --- |
|  | **UP SHELF** | **UP SHORE** | **CGI** | **DW SHORE** | **DW SHELF** | **^1^Promoter** |  | **Exon** | **CDS** | **^2^Repr. Transcript region** |  |
| Human | 284,198 | 445,562 | 1,599,039 | 433,223 | 282,248 | 912,348 |  | 1,157,258 | 500,347 | 2,288,144 | 3,138,749 |
| AGM (HPR) | 246,970 | 554,839 | 747,884 | 555,192 | 248,410 | 326,734 |  | 363,239 | 265,598 | 800,265 | 1,657,641 |
| AGM (+OPR) | 249,682 | 559,025 | 752,032 | 559,519 | 250,581 | 349,499 |  | 364,867 | 266,588 | 809,122 | 1,680,406 |
| CM (HPR) | 326,932 | 661,039 | 796,929 | 657,577 | 328,502 | 305,570 |  | 672,437 | 339,605 | 1,230,949 | 1,808,794 |
| CM (+OPR) | 327,718 | 663,014 | 798,668 | 659,715 | 329,010 | 309,205 |  | 673,450 | 340,109 | 1,232,571 | 1,812,429 |

**^1^**Promoter: Up 2 kb from TSS (transcription start site).

^2^Repr. Transcript region: For definition of TSS, we redefined the longest one among transcripts having same gene symbol.
