# Supplementary material for: Successful application of human-based methyl capture sequencing for methylome analysis in non-human primate models
Source: BMC Genomics. 2018 Apr 18;19:267. doi: 10.1186/s12864-018-4666-1 (PMC5907189; doi:10.1186/s12864-018-4666-1)
Supplement: Supplementary file 3 — Table S3. Summary of alignment statistics about sequenced reads. (DOCX 28 kb) [file 12864_2018_4666_MOESM3_ESM.docx]

Table S3. Summary of alignment statistics about sequenced reads.

| **Species** | **ID** | **Raw reads** | **Mapped reads** | **Uniquely mapped**  **reads** | **Mapping efficiency (%)** | **Duplicate (%)** | **Deduplicated**  **reads** |
| --- | --- | --- | --- | --- | --- | --- | --- |
| AGM | A01 | 82,042,708 | 66,456,932 | 65,406,636 | 79.72 | 5.74 | 61,654,018 |
|  | A02 | 79,989,134 | 68,162,908 | 67,567,108 | 84.47 | 18.29 | 55,211,440 |
|  | A03 | 164,286,834 | 68,407,310 | 67,539,506 | 41.11 | 40.82 | 39,972,142 |
| CM | C01 | 81,937,502 | 62,120,452 | 61,159,528 | 74.64 | 24.07 | 46,435,720 |
|  | C02 | 81,189,620 | 59,698,958 | 58,753,566 | 72.37 | 29.04 | 41,690,966 |
|  | C03 | 84,561,170 | 63,696,064 | 62,806,502 | 74.27 | 25.59 | 46,733,796 |
|  | C04 | 84,583,766 | 63,596,056 | 62,720,040 | 74.15 | 20.52 | 49,850,882 |
|  | C05 | 81,150,086 | 60,653,068 | 59,735,658 | 73.61 | 26.37 | 43,983,534 |
|  | C06 | 80,090,882 | 58,316,756 | 57,487,524 | 71.78 | 24.18 | 43,585,256 |
|  | C07 | 85,283,860 | 63,769,602 | 62,872,066 | 73.72 | 29.41 | 44,383,212 |
|  | C08 | 81,788,464 | 60,073,724 | 59,058,538 | 72.21 | 21.85 | 46,153,506 |
|  | C09 | 82,851,484 | 62,040,720 | 61,111,138 | 73.76 | 20.80 | 48,399,192 |
|  | C10 | 164,543,162 | 69,642,192 | 68,418,466 | 41.58 | 23.37 | 52,425,886 |
|  | C11 | 79,836,782 | 56,544,190 | 55,494,782 | 69.51 | 30.46 | 38,592,114 |
|  | C12 | 79,409,362 | 58,200,474 | 57,105,212 | 71.91 | 29.54 | 40,233,780 |
|  | C13 | 86,800,236 | 64,329,316 | 63,312,700 | 72.94 | 37.59 | 39,512,228 |
