# Supplementary material for: Successful application of human-based methyl capture sequencing for methylome analysis in non-human primate models
Source: BMC Genomics. 2018 Apr 18;19:267. doi: 10.1186/s12864-018-4666-1 (PMC5907189; doi:10.1186/s12864-018-4666-1)
Supplement: Supplementary file 4 — Table S4. On-targeted reads and average depth with accumulative depth coverage on CG sites. (DOCX 31 kb) [file 12864_2018_4666_MOESM4_ESM.docx]

Table S4. On-targeted reads and average depth with accumulative depth coverage on CG sites.

| **Species**  **(Target)** | **ID** | **On-targeted reads** | **Average depth (X)** | **Coverage of accumulative depth (%)** | | | | |
| --- | --- | --- | --- | --- | --- | --- | --- | --- |
|  |  |  |  | **≥1X** | **≥2X** | **≥5X** | **≥10X** | **≥30X** |
| AGM  (HPR) | A01 | 35,853,194 | 59.86 | 97.88 | 96.89 | 93.74 | 88.08 | 76.07 |
|  | A02 | 32,180,073 | 53.33 | 97.88 | 96.82 | 93.36 | 87.07 | 73.73 |
|  | A03 | 20,246,169 | 33.20 | 97.51 | 95.87 | 90.32 | 79.88 | 59.46 |
| AGM  (+OPR) | A01 | 35,911,339 | 57.90 | 95.08 | 93.91 | 90.71 | 85.20 | 73.56 |
|  | A02 | 32,232,924 | 51.58 | 95.00 | 93.79 | 90.34 | 84.23 | 71.31 |
|  | A03 | 20,284,661 | 32.12 | 94.85 | 92.98 | 87.44 | 77.29 | 57.52 |
| CM  (HPR) | C01 | 27,870,425 | 48.39 | 98.35 | 97.23 | 93.25 | 85.87 | 70.72 |
|  | C02 | 25,585,415 | 45.17 | 98.11 | 96.88 | 92.49 | 84.57 | 68.59 |
|  | C03 | 28,989,871 | 50.59 | 98.40 | 97.35 | 93.62 | 86.72 | 72.23 |
|  | C04 | 30,623,745 | 54.03 | 98.42 | 97.41 | 93.84 | 87.32 | 73.73 |
|  | C05 | 26,858,588 | 46.80 | 98.28 | 97.14 | 92.96 | 85.36 | 69.78 |
|  | C06 | 26,981,422 | 47.58 | 98.25 | 97.11 | 93.01 | 85.55 | 70.20 |
|  | C07 | 27,393,583 | 48.41 | 98.34 | 97.24 | 93.29 | 86.07 | 70.94 |
|  | C08 | 27,699,890 | 48.88 | 98.25 | 97.10 | 93.00 | 85.64 | 70.71 |
|  | C09 | 29,256,790 | 51.52 | 98.39 | 97.36 | 93.61 | 86.80 | 72.56 |
|  | C10 | 30,524,949 | 45.72 | 98.64 | 97.63 | 94.09 | 87.07 | 71.39 |
|  | C11 | 23,267,351 | 41.66 | 97.96 | 96.63 | 91.82 | 83.12 | 65.94 |
|  | C12 | 24,361,414 | 43.04 | 98.09 | 96.81 | 92.20 | 83.90 | 67.13 |
|  | C13 | 24,130,093 | 42.45 | 98.08 | 96.77 | 92.05 | 83.62 | 66.67 |
| CM  (+OPR) | C01 | 27,887,929 | 48.32 | 98.25 | 97.13 | 93.13 | 85.75 | 70.62 |
|  | C02 | 25,601,507 | 45.11 | 98.02 | 96.78 | 92.37 | 84.46 | 68.49 |
|  | C03 | 29,008,461 | 50.53 | 98.30 | 97.25 | 93.51 | 86.61 | 72.13 |
|  | C04 | 30,643,083 | 53.95 | 98.33 | 97.31 | 93.73 | 87.20 | 73.62 |
|  | C05 | 26,875,356 | 46.73 | 98.19 | 97.03 | 92.84 | 85.24 | 69.68 |
|  | C06 | 26,998,273 | 47.51 | 98.15 | 97.00 | 92.89 | 85.44 | 70.11 |
|  | C07 | 27,411,254 | 48.34 | 98.24 | 97.13 | 93.17 | 85.95 | 70.84 |
|  | C08 | 27,717,569 | 48.81 | 98.16 | 97.00 | 92.88 | 85.53 | 70.61 |
|  | C09 | 29,275,403 | 51.45 | 98.30 | 97.25 | 93.49 | 86.68 | 72.46 |
|  | C10 | 30,545,844 | 45.66 | 98.54 | 97.52 | 93.97 | 86.95 | 71.29 |
|  | C11 | 23,282,156 | 41.60 | 97.86 | 96.52 | 91.70 | 83.01 | 65.84 |
|  | C12 | 24,376,715 | 42.98 | 98.00 | 96.70 | 92.09 | 83.78 | 67.03 |
|  | C13 | 24,145,455 | 42.39 | 97.98 | 96.67 | 91.93 | 83.50 | 66.58 |
