# Supplementary material for: Successful application of human-based methyl capture sequencing for methylome analysis in non-human primate models
Source: BMC Genomics. 2018 Apr 18;19:267. doi: 10.1186/s12864-018-4666-1 (PMC5907189; doi:10.1186/s12864-018-4666-1)
Supplement: Supplementary file 5 — Table S5. Average methylation level on CG sites for each genomic regions (mean ± s.d.). (DOCX 27 kb) [file 12864_2018_4666_MOESM5_ESM.docx]

Table S5. Average methylation level on CG sites for each genomic regions (mean±s.d.).

| **Species**  **(Target)** | **Avg. met. level** | **Regulatory region** | | | | | |  | **Intragenic region** | | |
| --- | --- | --- | --- | --- | --- | --- | --- | --- | --- | --- | --- |
|  |  | **UP**  **SHELF** | **UP**  **SHORE** | **CGI** | **DW**  **SHORE** | **DW**  **SHELF** | **^1^Promoter** |  | **EXON** | **CDS** | **^2^Repr. Transcript region** |
| AGM  (HPR) | 36.8 ± 0.27 | 38.9 ± 0.23 | 25.0 ± 0.45 | 14.1 ± 0.57 | 24.5 ± 0.45 | 37.9 ±0.20 | 17.4 ± 0.55 |  | 46.9 ± 0.22 | 55.2 ± 0.17 | 46.2 ± 0.16 |
| AGM  (+OPR) | 36.8 ± 0.28 | 38.8 ± 0.24 | 25.0 ± 0.45 | 14.1 ± 0.57 | 24.4 ± 0.45 | 37.9 ± 0.20 | 17.9 ± 0.59 |  | 46.8 ± 0.21 | 55.2 ± 0.15 | 46.2 ± 0.16 |
| CM  (HPR) | 37.0 ± 0.66 | 37.5 ± 0.71 | 25.5 ± 0.62 | 14.8 ± 0.46 | 25.5 ± 0.64 | 37.3 ± 0.69 | 16.8 ± 0.54 |  | 38.9 ± 0.58 | 51.7± 0.66 | 40.4 ± 0.66 |
| CM  (+OPR) | 37.0 ± 0.66 | 37.4 ± 0.71 | 25.5 ± 0.62 | 14.8 ± 0.46 | 25.5 ± 0.64 | 37.2 ± 0.69 | 16.9 ± 0.54 |  | 38.6 ± 0.58 | 51.6 ± 0.66 | 40.4 ± 0.66 |

**^1^**Promoter: Up 2 kb from TSS (transcription start site).

^2^Repr. Transcript region: For definition of TSS, we redefined the longest one among transcripts having same gene symbol.
